# Leveraging the Elder Mistreatment Emergency Department Toolkit to Meet CMS’s Age-Friendly Hospital Measures

**DOI:** 10.1093/geroni/igaf122.4387

**Published:** 2025-12-31

**Authors:** Ruthann Froberg, Scott Bane, Kristin Lees Haggerty

**Affiliations:** Education Development Center, Waltham, Massachusetts, United States; The John A. Hartford Foundation, New York CIty, New York, United States; Education Development Center, Waltham, Massachusetts, United States

## Abstract

This year, the Centers for Medicare & Medicaid Services’ (CMS) introduced an Age-Friendly Health Systems (AFHS) Hospital Measure to assess hospital commitment to providing care to older adult patients. The free, publicly available Elder Mistreatment Emergency Department (EMED) Toolkit—developed by the National Collaboratory to Address Elder Mistreatment—provides a system intervention that aligns with two of this Measure’s domains: social vulnerability and age-friendly care leadership. Utilizing the EMED Toolkit to address this new measure is an opportunity to integrate routine identification of elder mistreatment, robust tracking of referrals for positive screens, and strategic leadership to support these efforts. Beyond its validated screening instrument, the Toolkit integrates decision-support prompts, staff workflows, and community-referral pathways into standard hospital processes, ensuring that social-vulnerability assessments trigger evaluation and service linkage. This presentation will provide a detailed crosswalk of the Toolkit that demonstrates functions corresponding to CMS’s social vulnerability and age-friendly care leadership attestations. The presentation will also include examples demonstrating the EMED Toolkit’s adaptability to different healthcare settings, offering health systems a scalable solution. This free resource provides a roadmap to assist health systems in meeting requirements of the AFHS measure and provides a framework to develop a multidisciplinary response team, ultimately elevating the care provided to older adults.

